# Does a parental history of cancer moderate the associations between impaired health status in parents and psychosocial problems in teenagers: a HUNT study

**DOI:** 10.1002/cam4.245

**Published:** 2014-04-10

**Authors:** Elisabeth Jeppesen, Ingvar Bjelland, Sophie D Fosså, Jon H Loge, Øystein Sørebø, Alv A Dahl

**Affiliations:** 1 National Resource Center for Late Effects, Department of Oncology, Oslo University Hospital, RadiumhospitaletOslo, Norway; 2 University of OsloOslo, Norway; 3 Department of Child and Adolescent Psychiatry, Haukeland University HospitalBergen, Norway; 4 Department of Clinical Medicine, Faculty of Medicine and Dentistry, University of BergenBergen, Norway; 5 School of Business and Social Sciences, Buskerud University CollegeHønefoss, Norway

**Keywords:** HUNT-2, parental cancer, psychosocial problems, teenagers, young-HUNT

## Abstract

Severe disease in a parent is associated with increased psychosocial problems in their children. However, moderating factors of such associations are less studied. In this cross-sectional population-based controlled study we examined the moderating effects of a history of parental cancer on the association between impaired health status in parents and psychosocial problems among their teenagers. Among families with both parents responding to the adult Health Survey of Nord-Trøndelag County of Norway (the HUNT-2 study) 71 couples were identified with primary invasive cancer in one parent. Their 81 teenage children took part in the Young-HUNT study. These families were compared to 322 cancer-free families with 328 teenagers. Based on self-report data the relations between three variables of parental impaired health and six psychosocial problems in teenagers were analyzed family wise by structural equation modeling. Significant associations between parental and teenagers' variables were observed in eight of 18 models. A history of parental cancer was a significant moderator which decreased four of eight significant associations. Such a history significantly weakened the associations between parental poor self-rated health and teenagers' anxiety/depression and school problems. A similar association of a history of parental cancer was found between psychological distress in parents and teenagers' feelings of loneliness and poor self-rated health. This study confirmed strong associations between impaired parental health and psychosocial problems in their teenagers. A history of parental cancer weakened several of the significant associations between parental impaired health variables and psychosocial problems in their teenagers.

## Introduction

Children of parents with both chronic and serious medical conditions (CMCs) including cancer show significantly more psychosocial problems than children of CMCs-free families 1–3. However, a diagnosis of CMCs does not capture the general health status of the sick parent, and should be supplemented by, for example, self-rated health which is an accepted measure of subjective health perception including both somatic and mental health problems 4.

Many studies based mostly on trauma theory have shown a significant direct association between parental cancer and psychosocial problems in teenagers such as anxiety/depression, confusion, sadness, anger, feelings of uncertainty, and loneliness 5–7. Feelings of uncertainty and loneliness also seem to be the emotions strongly associated with teenagers emotional and behavioral functioning and decreased over time 8. According to findings from previous studies, time since diagnosis appeared to be unrelated to stress response symptoms in teenagers (1–5 years after diagnosis) 9–11. These findings suggest that parental cancer may be a traumatic stressor of variable duration.

In contrast, some studies based on resilience theory 12 have considered occurrence of parental cancer as an ordinary negative life event for the family, and these studies mostly report a nonsignificant association between parental cancer and psychosocial problems in children and teenagers 13–15. Our group recently published such a study showing no significant differences of psychosocial problems between teenagers with a history of parental cancer in their families and teenage controls from cancer-free families 15.

Studies of the relation between parental cancer and psychosocial problems in teenagers regularly lack control groups, and the effects of moderator variables have hardly been investigated. The Health Study of Nord-Trøndelag County of Norway (HUNT study) 16 allowed us to examine whether a history of parental cancer moderated the associations between indicators of impaired health status of parents (adult HUNT-2 study) and psychosocial problems in their teenagers (Young-HUNT study). In this study, a history of parental cancer was treated as a family characteristic variable. On the basis of our previous study we hypothesized that such a history would have small effect on the association between impaired parental health status and psychosocial problems in their teenagers.

## Methods

### Organization of the surveys

The population of Nord-Trøndelag County in Central Norway has been examined through several health surveys (the HUNT surveys) [www.ntnu/hunt]. The County is fairly representative of Norway as a whole concerning demographic and health variables 16.

The Young-HUNT survey (1998–2000) comprised of all students in the County registered in junior high and high schools (13–19 years). They were invited to participate in a study arranged during school hours. Information was collected by a questionnaire and anthropometric examinations. Among 10,202 students invited, 8986 (88%) participated. The HUNT-2 study (1995–1997) invited by letter 92,936 adults ≥20 years to participate and 65,220 (70%) returned the questionnaire attached.

### Identification of parents with cancer

Due to the unique identity number of all individuals living in Norway, linkages between several national databases were possible. The Young-HUNT participants were linked with their parents, and based on identity numbers parents with a cancer diagnosis were identified within the Cancer Registry of Norway (CRN) 17.

Since 1953 the CRN has collected medical and demographic data on all new cases of cancer in Norway based on statutory registration requirements. The incidence data of new cancers are close to complete 17, while data on treatments and relapse are less complete, and therefore, not used in this study.

Only primary invasive malignancies were included, excluding basocellular carcinomas of the skin. Furthermore, the date of their primary cancer diagnosis had to precede the date of the Young-HUNT participation of their teenagers, and the parents had to be alive at that date. Details on type of cancer and time since primary diagnosis were retrieved for the relevant parents.

### The study sample

#### Cancer families

We included 71 families where both parents were living together, both had responded to the HUNT-2 and one of them was registered with a cancer diagnosis (35 fathers and 36 mothers). These families had 81 teenagers who took part in the Young-HUNT study, and they represented a subsample of teenagers of parents with cancer studied previously 15. Eight families had two teenagers and one family had three, and five of the parents had been diagnosed with cancer before their teenager was born.

#### Cancer-free families

Among 429 matched control teenagers studied previously 15, 328 belonged to 322 families where both parents were living together and also had taken part in the adult HUNT-2 study. Six of these families had two participating teenagers.

### Young-HUNT and HUNT-2 questionnaires

The Young-HUNT questionnaire covered physical and mental health, lifestyle, and socio-demographic information. Based on the literature 3 six variables were selected as indicators of psychosocial problems in teenagers (Table1). The HUNT-2 contained a questionnaire covering the same information completed by the parents. Based on previous studies three variables were selected as indicators of impaired health status in parents (Table2). Importantly, our definition of “severe medical condition” excluded cancer.

**Table 1:** Definitions of psychosocial problem variables based on the Young-HUNT questionnaire.

Definitions of psychosocial teenager (Young-HUNT study) variables			
Problem	Definition	Dichotomization	Reference
Somatic stress symptoms	Headache, pains in neck/shoulders, pain in joints/muscles, stomach pain, nausea, constipation, diarrhea, palpitations during last 12 months	0: 0–1 symptom 1: ≥2 symptoms	
Feeling lonely		0: sometimes/seldom/never 1: very often/often	
Eating problems	7-item version of the Eating Attitude Test (EAT-7) with sum score from 0 to 14	0: no problem (score 0–4) 1: problem (score >4)	Bjornelv et al. 28
Anxiety/depression	Symptom Check List 5 version (SCL-5) with four response alternatives, mean sum score ranges from 1 to 4	1: caseness of anxiety/depression mean score ≥2	Strand et al. 29
School problems	Three dimensions of school adjustment related to various experiences at school: Attention (three items), conduct (four items), and dissatisfaction (four items) with four response alternatives, ranging from “never” to “very often”	Low or high scores accordingly: attention problems >7; conduct problems >8; dissatisfaction >11 0: no school problems 1: ≥1 school problems present	Torvik et al. 30
Poor self-rated health	How would you say that your health is currently?	0: very good/good 1: not so good/poor	Cuypers et al. 31

**Table 2:** Definitions of variables indicating parents impaired health status (the adult HUNT-2 study).

Variables	Definition	Dichotomization	Reference
Severe medical condition	Were to the question “Do you have or had asthma, myocardial infarction, angina, stroke or diabetes?”	0: no 1: one or more positive responses	
Poor self-rated health	How would you say that your health is currently?	0: very good/good 1: not so good/poor	Cuypers et al. 31
Psychological distress	*The Hospital Anxiety and Depression Scale (HADS)*. The HADS consists of 14 items, 7 on the depression subscale (HADS-D) and 7 on the anxiety subscale (HADS-A). Each item is scored on a 4-point scale from 0 (not present) to 3 (considerable). We used the HADS Total score (Spearman's coefficient *ρ* 0.60–0.70).	Total score from 0 (minimum symptom load) to 42 (maximum symptom load)	Bjelland et al. 23

### Design and statistical considerations

This cross-sectional family survey examined the associations between indicators of impaired health status in parents from 71 cancer families and 322 noncancer families and psychosocial problems among 409 teenagers of these families. Specified structural equation models (SEMs) tested the associations between three parental-health variables and six teenager-psychosocial variables, altogether 18 models. Tests were done to uncover to what degree each model was moderated by the presence (score = 1) or absence (score = 0) of a history of parental cancer in the family. In addition, we tested if such a history had a direct effect on the teenager-psychosocial variables.

**Figure 1:**
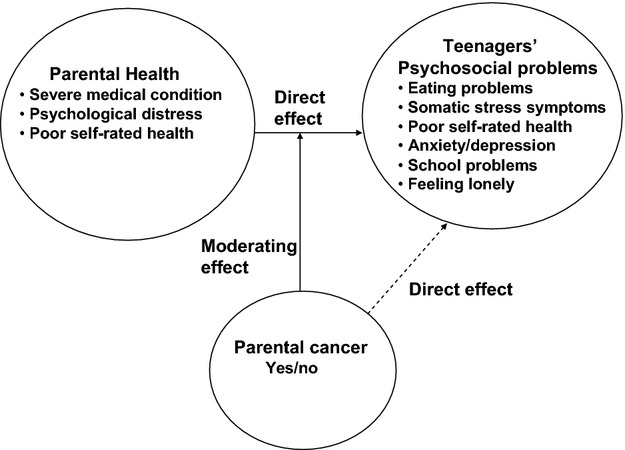
The model for examining associations between parents impaired health status and psychosocial problems in teenagers using presence or absence of a history of parental cancer as a moderator.

Categorical variables were analyzed using Fisher's exact test. Continuous variables were analyzed with *t*-tests and time since diagnosis of cancer with Mann–Whitney *U* test. The strength of associations between time since diagnosis, gender, and age of parents with cancer and the psychosocial variables in teenagers, was estimated by binary logistic regression with odds ratios (ORs) and 95% confidence intervals (95% CI). The internal consistencies of scales were examined with Cronbach's coefficient *α*. The correlations between each of the indicators of parental impaired health status and each of the teenagers' psychosocial problems were tested with Spearman's rank correlation test (coefficient *ρ*). Since only 16 of 409 teenagers were siblings, they were handled statistically as nonrelated individuals. These statistical analyses were performed by using Predictive Analytics SoftWare (PASW) for PC version 18 delivered by the IBM Corporation, New York, NY. The Product Indicator Approach 18 was utilized to model parental cancer as a moderator. Partial Least Squares Path Modeling (PLS-PM) with XlStat was used as the tool for modeling the moderator effect. PLS-PM is a statistical approach for SEMs of observed (e.g. presence of cancer) and latent (e.g. score of The Hospital Anxiety and Depression Scale [HADS]) variables. A Monte Carlo simulation performed by Chin and Newsted (1999) indicated that PLS can be performed with a sample size as low as 50 19. Group comparisons, that is comparisons of model estimates for different groups of observations (e.g. control parents vs. parents with cancer), can be regarded as a special case of moderating effects 20. The categorical moderator variable (presence or absence of a history of parental cancer) is assumed to influence the strength of the direct effect between the independent variables (indicators of impaired health status) and the dependent variable (each of the teenagers' psychosocial problems) (Fig.1).

The strengths of the relationships were expressed as standardized regression coefficients (sometimes referred to as *β* coefficients), with a range from −1 to +1. Such coefficients allow the direct comparison of the relative influence of, for example, parental health variables on teenager psychosocial variables, in addition to the relative influence of a history of parental cancer on the relationship between the two aforementioned variables. Significant coefficients were identified by *t*-values (i.e. 1.96 or larger). Because of the very limited number of studies with a similar design, we consider this study as explorative, and the significance level was, therefore, set at *P* < 0.05.

### Ethics

The National Data Inspectorate and the Central Norway Regional Committee for Medical and Health Research Ethics approved the Young-HUNT and the HUNT-2 studies. All respondents gave written informed consent, except students younger than 16 years for whom the parents gave such consent. Linkage between the Young-HUNT participants and their parents was approved by the National Data Inspectorate. Approval of this study was given by the South-East-Norway Regional Committee for Medical and Health Research Ethics.

## Results

### Characteristics of the parents

Family characteristics are shown in Table3. Among the parents with cancer the mean age was 45 years (mothers) and 48 years (fathers). The types of cancer were melanomas (23%), breast (22%), testicular (14%), thyroid (8%), gynecological (8%), urinary (8%), leukemia (6%), and others (11%). The median time since diagnosis of cancer in fathers was 48 months (range 1–257) and in mothers 61 months (range 1–329) (*P* = 0.76). There were no significant associations between time since cancer diagnosis and any of the six psychosocial problems in the teenagers.

**Table 3:** Family characteristics of parents.

Parents	Cancer families				Cancer-free families	
	Fathers with a history of cancer		Mothers with a history of cancer		Fathers and mothers	
	Fathers *n* = 35	Spouses *n* = 35 (mothers)	Mothers *n* = 36	Spouses *n* = 36 (fathers)	Fathers *n* = 322	Mothers *n* = 322
Age, mean (SD)	48 (7.2)	46 (6.1)	45 (5.9)	46 (5.6)	46 (5.7)	43 (5.2)
Level of basic education, *N* (%)						
<13 years	26 (74)	25 (74)	22 (63)	22 (61)	240 (76)	232 (73)
≥13 years	9 (26)	10 (26)	14 (37)	34 (39)	82 (24)	90 (27)
Work status, *N* (%)						
Employed	25 (71)	29 (83)	29 (81)	25 (71)	298 (73)	276 (65)
Unemployed	10 (29)	6 (17)	7 (19)	10 (29)	24 (27)	46 (35)
Severe medical condition, *N* (%)	6 (17)	3 (8)	0 (0)	4 (11)	41 (13)	26 (8)
Poor self-rated health, *N* (%)	6 (17)	8 (29)	6 (17)	4 (11)	56 (17)	55 (13)
Level of psychological distress, mean (SD)	8.4 (5.4)	7.3 (5.6)	7.8 (5.9)	7.3 (4.5)	7.8 (5.4)	7.7 (5.9)
No. of daughters	17		16		172	
No. of sons	20		28		156	

No differences between families with a history of cancer and cancer-free families were statistically significant (*P* < 0.05).

We did not observe any significant differences between parents in cancer families and parents in cancer-free families concerning age, socio-demographic variables, severe medical conditions, self-rated health, or psychological distress (Table3).

### Characteristics of the teenagers

Among the teenagers in the families affected by parental cancer there were 33 girls and 48 boys, with a median age of 16 years (range 13–19). Among teenagers from families without a history of parental cancer, we identified 156 girls and 172 boys, with a median age of 16 years (range 13–19). No significant differences were observed between the teenagers from cancer and cancer-free families concerning the six psychosocial problems (Table4). The time gap between parents' and teenagers' participation in their respective studies (HUNT-2 and Young-HUNT) ranged between 0 and 19.6 months.

**Table 4:** Family characteristics of teenagers.

Teenagers psychosocial problems	Teenagers			
	Case (*n* = 81)	Controls (*n* = 328)	*P*-value	OR
Eating problems	10 (12)	30 (9)	0.41	0.34–1.56
Feeling lonely	3 (4)	25 (8)	0.36	0.64–7.36
Poor self-rated health	3 (4)	28 (9)	0.17	0.72–8.22
Anxiety/depression	12 (15)	50 (16)	1.00	0.53–2.10
School problems	30 (37)	133 (41)	0.61	0.71–1.71
Somatic stress symptoms	29 (36)	107 (33)	0.60	0.53–1.14

Values are expressed as *N* (%). No differences between teenagers with a history of cancer in a parent and teenagers with cancer-free parents were statistically significant (*P* < 0.05).

### Internal consistencies of scales and correlations among variables

The internal consistencies (Cronbach's coefficient *α*) of the EAT-7 scale was 0.62, of the SCL-5 0.73, for school attention problems 0.63, conduct problem 0.68, and dissatisfaction at school 0.54, and of the HADS total 0.84. The correlations between the parental impaired health-related variables were a maximum of *ρ* = 0.32, and in particular the correlation between severe medical condition and self-rated health in parents was *ρ* = 0.11. Between the six psychosocial problems of the teenagers the maximum correlation coefficient was 0.32.

### The association between parents impaired health status and psychosocial problems in teenagers and the moderating effects of a history of parental cancer

There was a positive and significant standardized regression coefficient between *severe medical conditions* in parents and school problems in teenagers (*β *= 0.27, *P* < 0.05). A history of parental cancer did not significantly modify the relationship between severe medical conditions in parents and school problems in teenagers. Weak positive and significant direct effects of such a history were observed in the models of parental severe medical conditions and teenagers' anxiety/depression (*β* = 0.11, *P* < 0.05) and school problems (*β* = 0.06, *P* < 0.05, Table5).

**Table 5:** Associations between parental and teenager variables with a history of cancer as a moderator given as standardized *β* values.

Parental variables (exogenous)	Direct effect	Teenager psychosocial problems	Cancer—moderator effect	Cancer—direct effect
Severe medical conditions	0.09	Eating problems	−0.20	−0.00
	0.22	Feeling lonely	−0.17	−0.03
	0.06	Poor self-rated health	−0.07	−0.05
	0.21	Anxiety/depression	−0.21	0.11*
	0.27*	School problems	−0.13	0.06*
	0.06	Somatic stress symptoms	−0.10	0.02
Psychological distress	0.00	Eating problems	0.29	−0.25
	0.23***	Feeling lonely	−0.26**	0.12
	0.17**	Poor self-rated health	−0.18*	−0.08
	0.36*	Anxiety/depression	−0.27	0.19
	0.39**	School problems	−0.31	0.30
	0.14**	Somatic stress symptoms	−0.11	0.05
Poor self-rated health	0.13	Eating problems	0.00	−0.06
	0.10	Feeling lonely	0.19	−0.08
	0.06	Poor self-rated health	0.00	−0.06
	0.45*	Anxiety/depression	−0.34**	−0.10*
	0.40**	School problems	−0.29*	−0.13*
	−0.03	Somatic stress symptoms	0.02	−0.01

* *P* < 0.05,

** *P* < 0.01,

*** *P* < 0.001.

Positive and significant associations were observed between *psychological distress in parents* and higher levels of all teenagers' psychosocial problems except eating disorders. A history of parental cancer significantly weakened the relationship between psychological distress in parents and loneliness (*β* = −0.26, *P* < 0.01) and poor self-rated health in teenagers (*β* = −0.18, *P *<* *0.05) (Table5). No significant direct effects of a parental cancer history were observed in the parental distress models.

Positive and significant associations were also observed between *parental poor self-rated health* and anxiety and depression (*β* = 0.45, *P* < 0.05) and school problems (*β* = 0.40, *P* < 0.01) in teenagers. A history of parental cancer significantly weakened the relationship between parental poor self-rated health and anxiety/depression (*β *= −0.34, *P* < 0.01) and school problems (*β *= −0.29, *P* < 0.05) in teenagers. In these two models such a history also had weak direct significant negative effect (*β* = −0.06, *P* < 0.05 and *β* = −0.10, *P* < 0.05, respectively).

## Discussion

In line with findings from previous studies 1,2 we found significant direct associations between eight out of 18 models between impaired parental health status and psychosocial problems in teenagers. A history of parental cancer was a significant moderator decreasing four of eight significant associations. Our hypothesis that such a history would have small moderating effects on the associations between impaired parental health status and psychosocial problems in their teenagers was supported. We found four significant direct effects of a history of parental cancer on psychosocial variables in teenagers, but these effects were weak with an explained variance of maximum 2%, and they should, therefore, be interpreted with care.

Only two-parent families are included in this study. These families are a subset from a previous study including 143 teenagers of parents with cancer and 429 control teenagers of parents without cancer ignoring if the parents had participated in the HUNT-2 study 15. In that sample and the current subset with 81 teenagers of parents with cancer and 328 control teenagers, the same lack of significant differences between case and controls teenagers was observed. This finding with lack of significant differences among the teenager groups supports the small impact of parental cancer as a stressor on psychosocial problems in teenagers 12.

In accordance with previously reported CMC studies 1, we also observed that severe medical conditions (cancer omitted) in parents were associated with school problems in teenagers 21. A history of parental cancer in a family did not affect the association between severe medical conditions and psychosocial problems in teenagers. This finding indicates that the presence of severe medical conditions in families increases teenagers' school problems independent of whether or not there is a history of cancer in the family.

We observed no differences in levels of parental psychological distress (total HADS score 22,23) between case and control families or between genders. Previous studies have reported general higher level of psychological distress in long-term cancer survivors and their spouses than cancer-free controls 24. However, other studies have documented lower rate of depression in cancer patients when compared to other illnesses 24. We found significant associations between psychological distress in parents and higher levels of all teenagers' psychosocial problems studied, except eating disorders. Surprisingly, the history of parental cancer in families has a lower influence on teenagers feeling of loneliness and poor self-rated health.

How could the observed modifying effect of a history of parental cancer be understood? One explanation could be that such a history exerts a strong effect on the family atmosphere due to repeated reminders of cancer in daily life, weakening the significant associations between parental and teenagers variables. Alternatively, we only included two-parent families, and at the group level such families function significantly better than single-parent ones 13. In two-parent families the partner probably provides extra support and attention to child care compensating for the consequences of previous cancer of the spouse. Parents with cancer seem to spend more time with their children than parents in families without cancer 25. Finally, cancer patients and their families often receive considerable long-term support and care from other family members, their social network, and the health care system.

Such considerations are in accordance with a study from the Netherlands showing that families affected by parental cancer and actively coping with the illness had lower levels of stress and conflicts than control families. The cancer families were relatively more expressive, cohesive, organized, and social 26. Findings from qualitative research also support that in case of parental cancer the patients and their partners actively coping with cancer both parents try to optimize the family situation including protection of their children in order to make them feel as secure as possible 25.

### Strengths and limitations

A major strength of this study is the completeness of the CRN data concerning parental cancer within a restricted geographic area also included in health surveys of teenagers and their parents making selection bias among parents with cancer and their teenagers unlikely. Most studies have collected data close to the time of diagnosis and primary treatment, when the emotional turmoil in the family members reaches its peak level, while we have considerably extended time intervals between cancer diagnosis and the surveys.

Both the Young-HUNT and the adult HUNT-2 are cohort studies with high response rates, contrary to other studies addressing the effects of parental cancer upon their teenager offspring. For the statistical modeling we were able to include a sufficient number of complete families in which all parents and their teenagers have rated themselves on the relevant variables. The opportunity to analyze similar proportions of fathers and mothers with cancer is also considered a strong point.

Our results should be considered in light of some limitations. The group of parents with cancer was quite heterogeneous with respect to cancer type, treatment, time since diagnosis, and prognosis. However, the disease-related variables showed no significant associations with teenagers' psychosocial problems. Lack of direct information as to how the cancer was managed within the family or by the family members, is another limitation. Unfortunately, we lack information about the quality of the parent–teenager relationship or the level of family function 27,5. Since we did not have this information, we found the term “a history of parental cancer” in the family to be the most adequate one underlining the lack of important data in this regard. The time gap between parents' and teenagers' date of participation in the Young-HUNT and HUNT-2 could represent a limitation. We have calculated the time and we found four outliers. We do not consider these outliers to cause any systematic errors, or to be a serious limitation in the study.

Due to our sample sizes, we had to choose rather crude statistical models and could not present more advanced path analyses. The relatively small sample also infers a risk for type II statistical errors that could explain the lack of significant differences between parental and teenager groups.

## Conclusions

This study confirmed strong associations between impaired parental health and psychosocial problems in their teenagers. Our hypothesis that a history of parental cancer had small effects on these associations was supported. Such a history weakened several of the significant associations between parental impaired health variables and psychosocial problems in their teenagers.
